# Determinants of stage at diagnosis of HPV-related cancer including area deprivation and clinical factors

**DOI:** 10.1093/pubmed/fdaa246

**Published:** 2021-01-29

**Authors:** Rohini Chakravarthy, Sarah C Stallings, Digna R Velez Edwards, Sifang Kathy Zhao, Douglas Conway, J Sunil Rao, Melinda C Aldrich, Erin Kobetz, Consuelo H Wilkins

**Affiliations:** Vanderbilt University School of Medicine, Nashville, TN, USA; Department of Medicine, Division of Geriatrics, Vanderbilt University Medical Center, Nashville, TN, USA; Division of Quantitative Sciences, Department of Obstetrics and Gynecology, Vanderbilt University Medical Center, Nashville, TN, USA; Department of Biomedical Informatics, Vanderbilt University, Nashville, TN, USA; Institute for Medicine and Public Health, Vanderbilt Epidemiology Center, Vanderbilt University Medical Center, Nashville, TN, USA; Institute for Medicine and Public Health, Vanderbilt Epidemiology Center, Vanderbilt University Medical Center, Nashville, TN, USA; Vanderbilt Institute for Clinical and Translational Research, Vanderbilt University Medical Center, Nashville, TN, USA; Department of Public Health Sciences, University of Miami School of Medicine, Miami, FL, USA; Division of Biostatistics, University of Miami School of Medicine, Miami, FL, USA; Department of Thoracic Surgery, Vanderbilt University Medical Center, Nashville, TN, USA; Division of Genetic Medicine, Department of Medicine, Vanderbilt University Medical Center, Nashville, TN; Department of Public Health Sciences, University of Miami School of Medicine, Miami, FL, USA; Department of Medicine, University of Miami, Coral Gables, FL, USA; Department of Medicine, Division of Geriatrics, Vanderbilt University Medical Center, Nashville, TN, USA; Meharry-Vanderbilt Alliance, Nashville, TN, USA; Office of Health Equity, Vanderbilt University Medical Center, Nashville, TN, USA

**Keywords:** cancer, race, social determinants

## Abstract

**Background:**

Collecting social determinants of health in electronic health records is time-consuming. Meanwhile, an Area Deprivation Index (ADI) aggregates sociodemographic information from census data. The objective of this study was to ascertain whether ADI is associated with stage of human papillomavirus (HPV)-related cancer at diagnosis.

**Methods:**

We tested for the association between the stage of HPV-related cancer presentation and ADI as well as the association between stage and the value of each census-based measure using ordered logistic regression, adjusting for age, race and sex.

**Results:**

Among 3247 cases of HPV-related cancers presenting to an urban academic medical center, the average age at diagnosis was 57. The average stage at diagnosis was Surveillance, Epidemiology and End Results Stage 3. In the study population, 43% of patients were female and 87% were white. In this study population, there was no association between stage of HPV-related cancer presentation and either aggregate or individual census variables.

**Conclusions:**

These results may reflect insufficient sample size, a lack of socio-demographic diversity in our population, or suggest that simplifying social determinants of health into a single geocoded index is not a reliable surrogate for assessing a patient’s risk for HPV-related cancer.

## Introduction

It is well established that increasing awareness on social and environmental factors play a critical role in determining health and health-related outcomes.^1^^,^^2^ The World Health Organization defines social determinants of health as ‘the conditions in which people are born, grow, live, work and age that shape health’.^3^ This includes socioeconomic status, education, employment and environment among others.^3^ The Institute of Medicine, the Department of Health and Human Services,^4^ the Association of American Medical Colleges,^5^ and numerous other organizations, institutions and independent healthcare providers have highlighted the importance of routinely collecting social and environmental determinants of health in electronic health records since these factors are large contributors to health inequity.^6^^,^^7^ In addition, the transition to accountable care organizations through the Patient Protection and Affordable Care Act has incentivized healthcare systems to track and manage these social and behavioral determinants of health to reduce utilization.^7^

At the same time, there are challenges to incorporating the collection of this type of information at the patient level and at the scale required to support population level health. A variety of tools exist including the PRAPARE, AHCS and IOM tools.^8^ Recently, electronic health records have become more complex and are now able to capture social determinants of health (SDOH) data, and the Office of the National Coordinator is leading efforts to standardize practices around how data are captured from a technical standpoint.^8^^,^^9^ Nevertheless, most clinics still rely on clinical staff implementing surveys of individual patients, which is both time-consuming and resource intensive.^8^ The benefits of collecting social and environmental data from the patient must be weighed against the increased burden placed upon patients and providers by collecting such data.^10^ Accessing these data at scale using related, collective measures such as those in the census could benefit population health studies now and facilitate implementation of these recommendations.

To address concerns about the scalability and sustainability of collecting SDOH,^7^ we test the theory that census is a reliable predictor of health outcomes in a novel population. The first outcome we studied was human papillomavirus (HPV). HPV is associated with high burden of oropharyngeal and anogenital cancers.^11^ These are some of the most preventable cancers and yet disparities exist due to a variety of factors including differences in access to screening, socioeconomic status, race/ethnicity and genetics.^12^ SDOH such as educational attainment,^13^ income^14^ and insurance status^15^ have been associated with worse outcomes. These outcomes depend on a complex relationship of individual and neighborhood-level social determinants. Teasing out the differences between these effects can be challenging, even with multilevel modeling techniques.^16^ Census data contain several neighborhood-level variables that have been linked to cancer outcomes including: socioeconomic status,^12^ educational attainment.^13^^,^^17^^,^^18^ The ability to geocode patient addresses and link them to relevant community-level social and environmental data can provide invaluable information for researchers as well as providers about the individual’s community without lengthening the clinical encounter.^19^

The American Community Survey (ACS) provides an opportunity to collect information about social, environmental and housing characteristics that can have an impact on health and health outcomes. The ACS is a nationwide, random sample survey conducted annually by the United States Census Bureau. Individual responses are aggregated into estimates for several geographic entities and made publicly available. According to the United States Census Bureau, a census tract is a relatively permanent geographical subdivision that contains approximately 4000 people. These were outlined in the early 1900s as permanent delineations large enough to make statistical comparisons across groups. A census block is a smaller unit with a minimum of 1500 people, but there are concerns about protecting the privacy of individuals by collecting data at this level.^20^ In addition, fewer variables are collected at the census block level.^21^ For these reasons, census tracts are utilized in this study. In addition to individual census variables, several indices have been created to encompass multiple socioeconomic components, which have been associated with health outcomes. An Area Deprivation Index (ADI) can be calculated to determine the overall socioeconomic status of the neighborhood of a given patient.

While neighborhood-level indicators are not a substitute for individually collected variables, they have shown utility in predicting health outcomes.^22^ Deprivation indices that have been studied include the Singh ADI,^23^ Townsend Index,^24^ Social Vulnerability Index,^25^ and a number of locally developed metrics. The majority of previous literature relies on a deprivation index composed of 17 different markers developed by Singh et al. through a factor analysis of 1990 American Community Survey. These studies demonstrated correlation with health outcomes such as readmissions,^21^ diabetes prevalence,^26^ and mortality.^23^ This study utilizes a different ADI with six of the markers. This six-variable ADI measure utilized in this study was selected because it can be calculated efficiently for a broad population using publicly available code.^27^ Furthermore, this institution’s research enterprise had successfully integrated this with existing electronic health record data. Finally, it was selected for its flexibility as there is potential to apply the measure to a variety of health outcomes in the future. So far, this ADI has shown correlation with hospital length of stay and hospital utilization in the first year of life as well as pediatric emergency medical services utilization. ^28,29^ Meanwhile, other indices have not been as useful predictors. The European Deprivation Index did not predict time to treatment or time to diagnosis in a population of cancer patients.^28^ In another study, the Neighborhood Disadvantage Index was not significantly correlated with birthweight of infants born to adolescent mothers after adjusting for patient-specific factors.^29^ The mixed evidence for the value of a quantitative index support the need for greater agreement in population-based studies looking at the utility of measuring SDOH.

The objective of this study is to ascertain whether ADI is associated with stage of HPV-related cancer at diagnosis. In the literature, cancer stage at disease presentation is commonly used as a surrogate for access to preventative measures as patients without access will present at higher stages.^30–32^ In one study of the Singh Index, women in the most-deprived group were more than 30% more likely to die from cervical cancer than the least-deprived group.^33^ A study in Sweden using a locally developed neighborhood deprivation index found that the risk of cervical cancer morbidity and mortality was significantly higher for patients with high neighborhood deprivation scores even after adjusting for individual patient factors.^34^ The same group also found similar results for lung cancer^35^ and prostate cancer.^36^

HPV was selected as the first diagnosis to study because the university data repository already contained staging information and ADI but had not previously studied the relationship between the two. The institution had plans to assess additional populations depending on the results of this study.

## Methods

### Study population

This retrospective case–control study was conducted in study population, which consisted of all patients presenting with an HPV-related cancer to Vanderbilt University Medical Center from 2010 to 2020. Vanderbilt University Medical Center is a tertiary referral center and has a large catchment area across the Southeastern United States. Cancer patients were identified at Vanderbilt University Medical Center (VUMC) through its Research Derivative using a retrospective study design. The Research Derivative (RD) is a database derived from the VUMC clinical systems and restructured for research.^37^ Ethical approval was obtained from the Vanderbilt University Institutional Review Board.

**Fig. 1:**
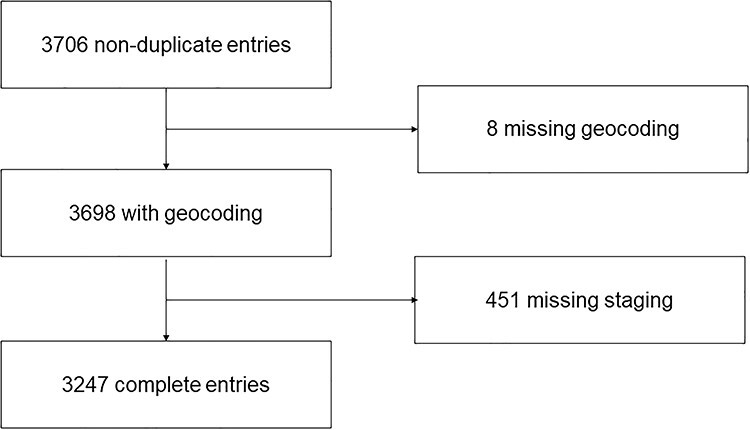
Schematic of patient inclusion criteria.

Subjects included in the analysis (1) carried at least two diagnostic codes (International Classification of Diseases-9 or International Classification of Diseases-10) for HPV-related cancers in the last 10 years, (2) had associated tumor registry information and (3) contained an address in the electronic health record, which could be geocoded to a census tract in Tennessee. Cancer diagnoses included in this study were as follows: anal, cervical, oropharyngeal, penile, vulvar and vaginal. There were 3706 patients in the RD with the requisite diagnostic codes in their electronic health record. From that starting set, 8 were excluded because of missing census information and 496 were excluded because they were missing the staging information from the National Tumor Registry. Age, race and gender were not predictive of missingness of cancer stage. The resulting population of 3247 individuals were included in the analysis (Fig. 1). Processed addresses were geocoded to census tracts using QGIS version 3.4.3 (Open Source Geospatial Foundation Project). Census variables were linked to clinical information derived from the RD and National Tumor Registry.

### Outcome

The outcome of interest was stage at presentation for HPV-related cancers as derived from tumor registry information. Surveillance, Epidemiology and End Results (SEER) stage guidelines were utilized for this study.^26^ Stages 8 (Benign) and 9 (Unknown) were excluded from analysis. Stage 6 is undefined.

### Covariates

Age-at-diagnosis was calculated from the date of birth and date of diagnosis as included in the cancer registry data. Sex was captured from cancer registry data and encoded as a binary variable with male as the reference. Race was captured from administratively assigned race in the electronic health record. Categories for race included White, Black, Indian or Alaska Native, Asian, Hispanic, other or unknown. Because of the low population of non-white participants, race was encoded as a binary variable (‘minority status’) for the purpose of analysis with White as the reference. Ethnicity (Hispanic, not Hispanic, unknown) could not be included in the model due to low prevalence of Hispanic participants (<1%).

Clinical information included SEER stage, age at diagnosis, race and other risk factors for HPV infection as indicated by diagnostic codes associated with patient encounters (prior sexually transmitted infections, presence of immunocompromising illness, HPV vaccination status, presence of drug or alcohol abuse). These clinical variables were selected *a posteriori,* based upon a review of relevant literature. These variables were selected as we believe they are associated with both ADI and HPV-related cancers. Number of sexual partners and tobacco use were not routinely collected in a standardized data format at the time of this study and therefore were not included in the model.

Because this was a retrospective study, individual variables such as individual income, educational qualification, employment status, family size, marital status and other variables were not available as these are not routinely collected in the electronic health record.

### ADI

An ADI is calculated from the 2015 United States 5-year American Community Survey linking the address listed for the patient’s residence in the electronic health record with the census tract group with the same area. This method was developed and validated by Brokamp et al. and is available publically.^38^ ADI for each included census tract was calculated.^38–40^ The components included in this calculation are as follows: (1) fraction of households with incomes below the poverty level in the last 12 months, (2) median household income in the past 12 months in 2015 inflation-adjusted dollars, (3) fraction of population 25 and older with educational attainment of at least high school graduation (includes GED equivalency), (4) fraction of population with no health insurance coverage, (5) fraction of household receiving public assistance income or food stamps or SNAP in the last 12 months and (6) fraction of vacant housing. A principal component analysis was applied and the first component was selected, which explains 60% of the variation in census tract-level measurements. This is was reduced to a single ‘deprivation index’ that ranges from 0 to 1 with a higher value representing a census tract with increased deprivation.^38^ In addition to modeling the effect of the overall ADI, models were also created with each of the individual components.

**Fig. 2:**
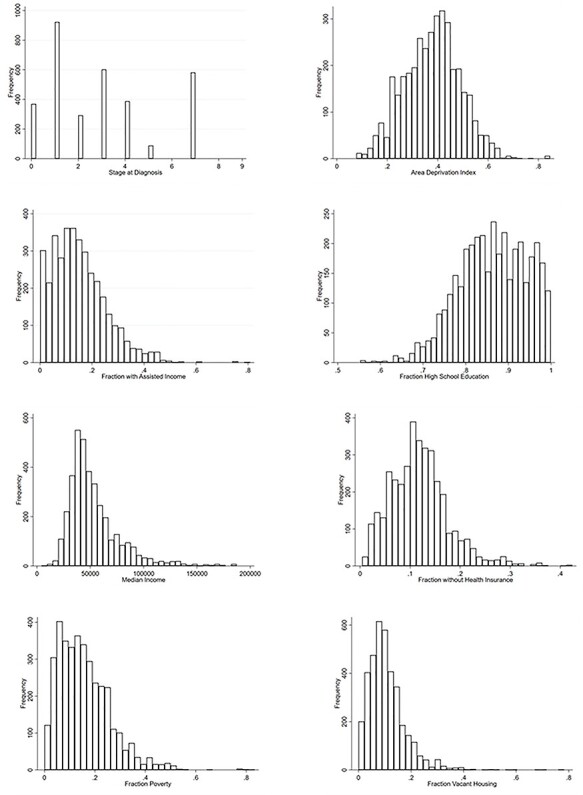
Frequency distribution of independent and dependent variables.

### Statistical analysis

Demographic characteristics and cancer stage were summarized using percentages, means and standard deviations. Ordinal logistic regression models were used to identify associations between a range of census tract variables with stage at diagnosis. This model was selected to predict the response of the ordinal variable to multiple inputs. The ordinal dependent variable was cancer stage at presentation (stage 0 to 7). A higher cancer stage increases the risk of a worse outcome. We constructed a separate model for each census variable. All models were adjusted for age at diagnosis, race and sex. Each model followed the following regression equation:

*Cancer Stage at Diagnosis = Census Variable + Age + Minority Status + Sex*

Analyses were summarized with odds ratios (OR) and 95% confidence intervals (CI). In addition, given previous studies showing the nonlinear relationship with ADI,^34^^,^^41^ we further examined this nonlinear relationship in this study population using ADI quartiles. Since observations of participants living in the same census tract may not be independent, we used robust estimate of variance to account for clustering within census tracts^28^^,^^42^ Secondary analyses tested for interactions between race and census track variables (ADI, median income and fraction with high school education) as well as sex and the same set of census tract variables using likelihood ratio tests. All analysis was conducted in STATA/SE version 15 (StataCorp LLC, College Station, Texas).

**Table 1:** Participant demographics

		*Total (%)*	*Stage 0*	*Stage 1*	*Stage 2*	*Stage 3*	*Stage 4*	*Stage 5*	*Stage 7*
Total		3247 (100%)	370 (12%)	923 (28%)	293 (9%)	602 (19%)	388 (12%)	89 (3%)	582 (18%)
Age									
	Missing	40 (1)	2	8	3	9	4	1	13
	<30	109 (3)	23	31	4	11	7	1	32
	30–40	220 (7)	45	74	18	27	15	3	38
	40–50	586 (18)	81	151	50	105	78	20	101
	50–60	937 (29)	108	232	86	216	130	19	146
	60–70	844 (26)	81	241	72	164	110	27	149
	70–80	381 (12)	22	137	43	62	36	11	70
	>80	130 (4)	8	49	17	8	8	7	33
Sex									
	Male	1836 (57)	160	487	150	431	246	50	312
	Female	1411 (42)	210	436	143	171	142	39	270
Race									
	Asian	33 (1)	6	11	0	3	5	0	8
	Black	261 (8)	40	67	30	34	28	8	54
	Hispanic	45 (1)	5	18	2	6	6	2	6
	Native American	6 (0)		3	0	0	0	1	2
	White	2822 (87)	312	813	256	544	336	75	486
	Other	9 (0)	3	2	0	2	1	0	1
	Missing	71 (2)	4	9	5	13	12	3	25
Ethnicity									
	Hispanic	45 (1)	5	18	2	6	6	2	6
	Not Hispanic	3115 (96)	361	890	284	583	372	84	541
	Unknown	87 (3)	4	15	7	13	10	3	35

**Table 2:** Census variables median (IQR)

	*Total*	*Stage 0*	*Stage 1*	*Stage 2*	*Stage 3*	*Stage 4*	*Stage 5*	*Stage 7*	*Missing*
Deprivation Index	0.38 (0.15)	0.38 (0.15)	0.38 (0.15)	0.39 (0.16)	0.38 (0.15)	0.39 (0.15)	0.39 (0.14)	0.38 (0.17)	0.39 (0.15)
Fraction assisted income	0.14 (0.13)	0.13 (0.14)	0.14 (0.13)	0.15 (0.14)	0.13 (0.14)	0.14 (0.13)	0.16 (0.12)	0.14 (0.15)	0.14 (0.13)
Fraction high school education	0.87 (0.12)	0.87 (0.11)	0.87 (0.12)	0.86 (0.11)	0.87 (0.11)	0.86 (0.12)	0.86 (0.10)	0.86 (0.13)	0.86 (0.13)
Median income	46875 (24825)	48007 (25392.5)	46944 (24475)	44329 (23228.25)	48250 (24967.25)	47539 (22897.25)	46819 (20581)	46762 (26594.5)	45589 (24552)
Fraction no health insurance	0.11 (0.07)	0.12 (0.07)	0.11 (0.07)	0.12 (0.07)	0.12 (0.07)	0.12 (0.07)	0.11 (0.07)	0.11 (0.07)	0.11 (0.07)
Fraction poverty	0.14 (0.14)	0.13 (0.15)	0.14 (0.14)	0.15 (0.14)	0.14 (0.14)	0.14 (0.13)	0.15 (0.16)	0.14 (0.14)	0.15 (0.13)
Fraction vacant housing	0.09 (0.08)	0.09 (0.07)	0.10 (0.08)	0.10 (0.07)	0.09 (0.08)	0.10 (0.07)	0.09 (0.08)	0.09 (0.08)	0.10 (0.09)

**Table 3:** Most frequent cancer types

*Cancer type*	*Frequency*
Tonsils	348
Rectum	304
Tongue	282
Cervix	182
Vulva	90
Penis	61
Anus	55
Rectosigmoid colon	52
Corpus uteri	51
Vagina	49

## Results

Among 3247 cases of HPV-related cancers, the average age at diagnosis was 57 (standard deviation: 13.5). The majority of patients were male (57%) and white (87%) (Table 1). The average ADI for the cohort was 0.38 [interquartile range (IQR): 0.15]. The average fraction of census tract with high school education was 0.87 (IQR: 0.12). The average census median income was $46,875 (IQR: $24,825). The distributions of individual and aggregate census markers are displayed in Table 2 and Fig. 2. The average stage at diagnosis was SEER Stage 3. The most frequent cancer locations were the tonsils, rectum and tongue (Table 3).

The majority of patients (78%) in this cohort reside in the state of Tennessee. According to the American Community Survey Factfinder, 48.7% of the state’s population identifies as male. Meanwhile, 78% of the state population identifies as white. For the 1490 census tracts in TN, the average ADI was calculated to be 0.42 (standard deviation: 0.12). Thus, a 0.1-unit change in deprivation index is equivalent to an effect size of 0.80 (i.e. 0.1/0.12). The average ADI weighted by population was calculated to be 0.40, suggesting that the most deprived individuals live in the most populous census tracts.

Ordinal logistic regression results are in Table 4. ADI was not significantly associated with stage at diagnosis (*P* > 0.05) after controlling for minority status, age-at-diagnosis, and sex. Each of the six components of the area deprivation index were also not found to be significantly associated with stage at diagnosis (*P* > 0.05). Furthermore, no particular quartile was significantly different from the remaining. Interaction terms between race and ADI, income, and education were not significant and therefore not included in the models. Similarly, interaction terms between sex and ADI, income, and education were not significant and therefore not included in the models. In addition, the variable clustering estimator analysis shows that stage of HPV-related cancer stage at diagnosis does not vary by ADI even after accounting for clustering within census tracts. Additional analysis at the county level also showed similarly insignificant results.

**Table 4:** Ordinal logistic regression results of relationship between patient characteristics and stage of presentation

	*Unadjusted*			*Adjusted*		
*Census variable description*	*OR*	*CI*	**P*-value*	*OR*	*CI*	**P*-value*
Deprivation Index	1.47	(0.84, 2.55)	0.18	1.40	(0.79, 2.48)	0.26
Fraction assisted income	1.59	(0.87, 2.89)	0.13	1.47	(0.78, 2.75)	0.23
Fraction high school education	0.56	(0.26, 1.20)	0.14	0.59	(0.27, 1.28)	0.18
Median income	1.00	(1.00, 1.00)	0.32	1.01	(1.00, 1.01)	0.36
Fraction no health insurance	1.37	(0.45, 4.17)	0.58	1.31	(0.42, 4.05)	0.64
Fraction poverty	1.38	(0.75, 2.51)	0.30	1.27	(0.68, 2.38)	0.44
Fraction vacant housing	1.70	(0.74, 3.89)	0.21	1.70	(0.75, 3.87)	0.20

The prevalence of risk factors (count of lifetime sexually transmitted infections, presence of immunocompromising illness, HPV vaccination status, presence of drug or alcohol abuse) in the dataset were too low to include in the analysis. We were not able to do additional sub-analysis by cancer type due to small numbers.

## Discussion

### Main finding

Based on previous studies of mortality in cervical cancer patients^33^^,^^34^, we predicted patients in neighborhoods with greater deprivation would present at higher stage-at-diagnosis. For this study population, ADI was not found to be significantly associated with stage at presentation in a cohort of HPV-related cancers.

### What is already known on this topic

Incorporating social and environmental factors into electronic health records is an issue with a variety of potential solutions.^4^^,^^7^^,^^8^^,^^19^ Census-based deprivation indices have been utilized in a variety of settings from predicting individual health outcomes^23^^,^^34–36^ to hospital utilization.^22^^,^^41^ Many studies have shown strong correlations while others have not.^24^^,^^29^ Different deprivation indices contain different subsets of census markers, however, demonstrating the lack of consensus on which variables are important for measuring socioeconomic characteristics. Census-based deprivation indices have been utilized in a variety of settings from predicting individual health outcomes to hospital utilization. Clearly, there is a need for agreement on what should be measured before we can determine the utility of such indices. These findings also raise the concern for publication bias around ADI.

For HPV-related cancers specifically, both Singh et al.^33^ as well as Li et al.^36^ found correlations between degree of deprivation and cervical cancer mortality. We were not able to replicate these results in our study of the broader HPV-related cancer population.

Our results could also be explained by the fact that neighborhood estimates are not a perfect substitute for measuring individual characteristics. An analysis of a small area deprivation measure in New Zealand demonstrated that even in the most deprived areas, 10% of individuals have none of the characteristics of deprivation, thereby demonstrating the poor correlation between these individuals and their corresponding neighborhood deprivation level.^42^ In contrast, a study that surveyed people individually about financial insecurity and housing stability found statistically significant correlations with health outcomes, specifically blood pressure and cholesterol levels.^43^ This suggests the need for increased screening at the individual level rather than reliance on geocoded estimates. Individual variables such as individual income, educational qualification, employment status, family size and marital status likely play an important and complex role in health outcomes and according to the results of this study are not adequately reflected in the calculated index.

Our differing results also reflect the complexity of SDOH. Epidemiologic studies alone have not been sufficient to address the causes of inequality. Research does not exist outside of the political, economic and cultural contexts.^44^ We recognize that a single index can oversimplify the complex biologic and epigenetic mechanisms that underlie these disparities.^45^

### What this study adds

Our findings are in contrast to previous studies, which have found correlations between other neighborhood deprivation indices and health outcomes.^23^^,^^41^^,^^46^ The inconsistency in our results compared to previous studies could be explained by variability in what is meant by socioeconomic deprivation, poor correlation between geocoded estimates and individual SDOH status, or insufficient statistical power. The results of this study question the use of ADI as a scalable and sustainable surrogate for collecting SDOH. According to our results, ADI did not show predictable and repeatable results in this population of patients at a tertiary care center. The results of our study suggest that geocoded indices may not be a reliable surrogate for capturing the complexity of underlying individual socio-demographic variables.

Strengths of this study are the fact that it takes advantage of routinely collected information and does not pose an additional burden to clinicians or patients. Another strength is this analysis incorporates disease-specific outcome rather than previous studies, which rely on hospital utilization data as a surrogate. This study model proposed here could be replicated in other disease-based cohorts. To eliminate variability in quality of treatment or hospital-based differences in care, this analysis was designed to utilize stage-at-diagnosis.

This method aims to support the conceptual framework provided by Hiatt and Breen, which suggests that cancer outcomes are a complex relationship between social determinants, biological factors and medical interventions. In this framework, social determinants refer to ‘physical and built environments that are part of or the result of human activity’. Examples included in this framework include occupation, income, education and health-insurance coverage. Outcomes are defined as those collected in cancer registries.^47^ By using publicly available census data, we hope to better understand the associations between these socioeconomic factors and HPV-related cancer outcomes.

### Limitations

It is unclear if the results of this study are generalizable to the general population. This study was conducted in a tertiary referral center in the South, which may not reflect the socio-demographic diversity of all clinical practices. The ADI is lower than the general population of Tennessee. Moreover, the study was conducted in a population with 90% of persons identifying as white, higher than the state-wide average, which limits the ability to accurately account for race as a covariate.

The statistical methods utilized in this analysis were not complex enough to account for measurement error associated with the census. The census polls a subset of the population to make estimates for a given census tract. These estimates are less reliable for census tracts with smaller populations. Since Tennessee is a predominantly rural state, this could lead to measurement error in our population. In addition, one could argue that variable clustering estimation does not adequately account for similarities between persons in the same census tract and that geospatial techniques might better account for this geographic proximity. Of note, census subdivisions were created for the purpose of the US Census not calculating SDOH and therefore may group dissimilar households. For these reasons, correlations between census variables and health outcomes are inconsistent.

In order to draw stronger conclusions about the general population, this study ought to be replicated in larger datasets with greater racial diversity. Because this was conducted as a retrospective chart review, we are unfortunately limited in the types of populations and variables that can be studied. Future studies should collect individual-level variables to compare these to the neighborhood-level factors. Additional disease-specific variables, such as sexual practices or smoking status would have enriched the findings of this study. Future studies of larger cohorts would also allow for the study of subgroups of disease populations and narrower socioeconomic subgroups. For example, studying the outcomes of oropharyngeal squamous cell carcinoma may be more informative than comparison across all HPV-related cancers. Alternatively, future iterations of this model could incorporate genetic ancestry to better account for biological differences rather and socially constructed perceptions of race. In addition, alternate geospatial techniques, such as geographically weighted regression analysis, could be considered to better account for geographic similarities and measurement error.

In conclusion, ADI is not associated with stage of HPV-related cancer at presentation. These results suggest that simplifying SDOH into a single geocoded index is not a reliable surrogate for assessing a patient’s risk for HPV-related cancer. These findings should be confirmed by larger studies that are more reflective of the socioeconomic characteristics of the general population.

## Notes

**Rohini Chakravarthy**, MD and MBA Candidate

**Sarah Stallings**, PhD, Research Assistant Professor

**Digna R. Velez Edwards**, PhD MS, Professor of Obstetrics and Gynecology, Director of Women's Health Research

**Sifang Kathy Zhao**, PhD Candidate

**Douglas Conway**, MS, Informatics Project Manager

**J. Sunil Rao**, PhD, Professor and Interim Chair of Public Health Services and Director

**Melinda Aldrich**, PhD MS, Associate Professor of Medicine and Thoracic Surgery and Bioinformatics

**Erin Kobetz**, PhD MPH, Associate Professor of Medicine, Public Health Sciences and Obstetrics/Gynecology, Director of Jay Weiss Institute for Health Equity at Sylvester Comprehensive Cancer Center, Senior Associate Dean for Health Disparities

**Consuelo H. Wilkins**, MD MSCI, Professor of Medicine and Vice President for Health Equity
